# Real-World Data on the Efficacy of Daratumumab in Patients with Relapsed/Refractory Multiple Myeloma and Amplification 1q

**DOI:** 10.3390/cancers17081261

**Published:** 2025-04-08

**Authors:** Magdalena Benda, Patrick Reimann, Elena Bletzacher, Axel Muendlein, Benda Bernhard, Bernd Hartmann, Minh Huynh, Klaus Gasser, Niklas Zojer, Theresia Lang, Georg Göbel, Jan-Paul Bohn, Stefan Schmidt, Eberhard Gunsilius, David Nachbaur, Emina Jukic, Maurus Locher, Ella Willenbacher, Wolfgang Willenbacher, Thomas Winder, Normann Steiner

**Affiliations:** 1 Department of Internal Medicine II, Feldkirch Academic Teaching Hospital, 6800 Feldkirch, Austria; magdalena.benda@lkhf.at (M.B.); patrick.reimann@lkhf.at (P.R.); bernd.hartmann@lkhf.at (B.H.); minh.huynh@lkhf.at (M.H.); klaus.gasser@lkhf.at (K.G.); niklas.zojer@lkhf.at (N.Z.); theresia.lang@lkhf.at (T.L.); thomas.winder@lkhf.at (T.W.); 2 Faculty of Medical Sciences, Private University of the Principality of Liechtenstein, 9459 Triesen, Liechtenstein; 3 Department of Internal Medicine V, Comprehensive Cancer Center Innsbruck, Medical University of Innsbruck, 6020 Innsbruck, Austria; elena.bletzacher@i-med.ac.at (E.B.); jan-paul.bohn@i-med.ac.at (J.-P.B.); stefan.schmidt@tirol-kliniken.at (S.S.); eberhard.gunsilius@tirol-kliniken.at (E.G.); david.nachbaur@tirol-kliniken.at (D.N.); ella.willenbacher@i-med.ac.at (E.W.); wolfgang.willenbacher@tirol-kliniken.at (W.W.); 4 Molecular Biology Laboratory, Vorarlberg Institute for Vascular Investigation and Treatment, 6850 Dornbirn, Austria; axel.muendlein@vivit.at; 5 Core Facility Internal Emergency and Intensive Care Medicine, University Clinic Innsbruck, 6020 Innsbruck, Austria; bernhard.benda@tirol-kliniken.at; 6 Department of Medical Statistics, Informatics and Health Economics, Medical University of Innsbruck, 6020 Innsbruck, Austria; georg.goebel@i-med.ac.at; 7 Institute of Human Genetics, Medical University of Innsbruck, 6020 Innsbruck, Austria; emina.jukic@i-med.ac.at (E.J.); maurus.locher@gmail.com (M.L.); 8 Department of Human Genetics, Inselspital Bern, University of Bern, 3010 Bern, Switzerland; 9 Syndena GmbH, Connect to Cure, 6020 Innsbruck, Austria; 10 University of Zurich, 8006 Zurich, Switzerland

**Keywords:** multiple myeloma, amplification 1q, cytogenetic high risk, daratumumab, CD38 antibody

## Abstract

**Background:** Treatment of multiple myeloma has advanced tremendously with the approval of anti-CD38 antibodies. Their efficacy is impressive but still controversial in the 1q amplification subgroup (amp1q). This retrospective study aims to provide real-world data. **Methods:** This trial is analyzing 74 patients with relapsed/refractory multiple myeloma treated with CD38Abs at the Medical University of Innsbruck (2016–2023). High-risk (HR) cytogenetics according to R-ISS (t(4;14), t(14;16), t(14;20), del(17p)), the presence of amp(1q21), the frequency of two HR markers (double hit), and the high-risk criteria agreed at IMS 2024 (HR-IMS24) were considered. **Results:** The median age of the 74 patients (62.1% male) was 62 years, with a median follow-up of six years. Most patients received third-line therapy (37.8%). R-ISS HR was documented in 39.2% of patients, double hit in 13.5% of patients, and HR-IMS24 in 32.4% of patients, while amp1q was detected in 35.1% of patients. The median OS was 66 months (35–89), and the median PFS was 17 months (6.5–26.9). While neither R-ISS HR nor isolated amp1q had an impact on progression-free survival (e.g., amp1q 7.03: 1.95–22.44; *p* = 0.347), the occurrence of a double-hit pattern significantly impaired PFS and OS (6.2: 1.4–16.4 months; *p* = 0.044; OS, 42.8: 25.9–74.6 months; *p* = 0.035). Patients fulfilling the HR-IMS24 criteria (32.4%, 24 patients) also exhibited an impaired PFS and OS (7: 2.7–18.1 months, *p* = 0.023; 40.12: 21.1–74.5 months, *p* = 0.01). **Conclusions:** This retrospective study highlights the durable effect of daratumumab on cytogenetic abnormalities, particularly amp1q. However, patients who meet the criteria for double-hit myeloma or the high-risk IMS2024 criteria remain a difficult-to-treat patient population who require early access to new treatment approaches.

## Simple Summary

The effect of daratumumab in gain/amp 1q patients has been much debated and questioned. CD38 antibodies are now the backbone of multiple myeloma therapy. The question of whether patients with gain 1q benefit from daratumumab is of interest in clinical practice and has been addressed in our work in a retrospective analysis of 74 patients in a relapsed/refractory setting. We found that the presence of gain 1q did not result in a significant difference in PFS or OS, whereas PFS and OS were significantly reduced when two high-risk markers were present (6.2: 1.4–16.4 months; *p* = 0.044; OS, 42.8: 25.9–74.6 months; *p* = 0.035) or IMS24 risk criteria were met (7: 2.7–18.1 months, *p* = 0.023; 40.12: 21.1–74.5 months, *p* = 0.01). According to our data, daratumumab can overcome the negative impact of gain/amp1q, but ultra-high-risk patients remain a difficult-to-treat cohort and require intensive treatments such as continuous treatment and T-cell-engaging therapies.

## 1. Introduction

The treatment of multiple myeloma has evolved significantly in recent years [1], with anti-CD38 antibodies (CD38Abs) being a key part of this encouraging development. In the Pollux and Castor studies, the efficacy of daratumumab was demonstrated, with overall response rates (ORRs) of 92.9% and 82.9% and a progression-free survival (PFS) of 45 and 16.7 months in relapsed and refractory disease, respectively, and it was approved for this indication in 2016 [2,3].

In 2019, the MAIA trial supported the approval of daratumumab in first-line settings for non-transplantable patients, finding a 47% reduction in the risk of progression or death in the DaraRd cohort [4].

Autologous stem cell transplantation (ASCT) remains the standard treatment for eligible patients, as confirmed by long-term data from multiple clinical trials [5].

Daratumumab has been introduced into the treatment of transplant-eligible patients on quadruple therapy. As expected following the initial data presented by Philipp Moreau for DaraVTd, the efficacy of the intensive DaraVRd treatment regimen has recently been demonstrated in the Phase III Perseus trial, with undeniable benefits in terms of durable response. A sustained MRD negativity of 63.9% was observed after 36 months of treatment, as well as a 58% reduction in the risk of disease progression or death while maintaining quality of life and a manageable safety profile [6].

Data on various quadruple regimens using the CD38 antibody isatuximab instead of daratumumab or replacing bortezomib with carfilzomib have added to the emerging picture. At the last EHA in 2024, long-term data from the IMROZ trial were presented on quadruple therapy in fit, non-transplantable patients [7,8,9,10].

Despite the impressive data, the increasing use of CD38-Ab is leading to an increase in the already costly treatment of multiple myeloma, especially in the maintenance setting [11].

The question of whether all patients should receive a quadruple regimen or whether it is possible that some patients could receive less-intensive therapy has been discussed repeatedly. In this context, amp1q particularly stands out. Amplification 1q has been detected in approximately 40% of patients and has been shown to have a negative prognostic value in repeated analyses, with frequent co-occurrence of other high-risk cytogenetic abnormalities [12]. The presence of two unfavorable cytogenetic markers is often referred to as double-hit myeloma. Amp1q with a copy number of ≥4 is now included in the second revised international staging system [13]. In 2024, the International Myeloma Society presented a new consensus on cytogenetic high risk, which includes the biallelic del(1p32) mutation in addition to the TP53 mutation/del(17p9). In addition, the combination of amp/gain1q and monoallelic del(1p32) and the combination of any one of the known translocations (t(4;14), t(14;16), t(14;20)) with either gain/amp1q and/or del(1p32) are considered to be cytogenetically high risk [14].

The benefit of daratumumab in high-risk patients has been critically discussed in subgroup analyses by authors such as Mohan et al., who demonstrated a PFS of 0.5 years (95% CI: 0.3–1.4 years) vs. 2.1 years (95% CI: 1.9 years-NR) [15]. This stands in contrast to the subgroup analyses of ICARIA, which demonstrated a PFS benefit with isatuximab in patients with isolated amp1q (11.2 vs. 4.6 months; hazard ratio, 0–50; 95% confidence interval (CI), 0.28–0.88) and in patients with amp1q regardless of the presence of other high-risk cytogenetic abnormalities (8.9 vs. 2.3 months; hazard ratio, 0.49; 95% CI, 0.24–0.99) [16].

Given the financial burden and the increasing use of CD38-Abs in first-line settings, the cost/benefit ratio in selected patient cohorts needs to be carefully evaluated to ensure limited resources are used responsibly and to continue to offer patients the best tailored treatment option.

The aim of this study is to analyze the response to CD38-Abs in terms of PFS, overall survival (OS), and duration of treatment (DOT) in clinical practice, considering different cytogenetic abnormalities. In particular, the influence of amp1q will be considered. To ensure the longest possible follow-up with a comparable/homogeneous patient population, patients in the refractory/relapsed phase were included from when approval was granted.

## 2. Methods

### 2.1. Objectives, Participants, and Oversights

This monocentric, single-arm, non-randomized retrospective data analysis included 74 patients with RRMM who were treated at the Innsbruck Medical University Hospital between 2016 and 2023. Patients were identified from the clinical information system. All patients received daratumumab as monotherapy or combination therapy. The main inclusion criteria were the use of daratumumab for more than one month and the presence of relapsed or refractory MM disease. All age groups, ethnicities, disease stages, and comorbidities were considered; however, the majority of the treated patients were of Western or Eastern European origin. The study was approved by the Ethics Review Board of the Medical University of Innsbruck—1181/2021. The dataset was anonymized, collected, and finalized in January 2023. Patients were followed up regularly, and their response to therapy was assessed according to the IMWG classification criteria. The classification was based on the Revised International Staging System (R-ISS) and the isotype of multiple myeloma. Data included the type and number of treatment regimens, next-generation sequencing, and survival analyses. The number of relapses before, during, and after daratumumab administration; the duration of daratumumab treatment; and cytogenetic aberrations at initial diagnosis and before and after daratumumab therapy were analyzed. PFS was defined as the time from the start of daratumumab treatment until relapse, last follow-up, or time of death. OS was calculated from the time of initial diagnosis until death. The overall response rate (ORR) was not calculated due to missing data. The extent of plasma cell infiltration into the bone marrow at the time of diagnosis and before and after daratumumab is shown.

### 2.2. Laboratory Analyses and Definition of Risk Criteria

Interphase FISH of the bone marrow was performed between January 2016 and June 2021 before the start of treatment with daratumumab. FISH analysis focused on the chromosomal regions 1p, 1q21, 11q22, 13q14, and 17p13. A FISH analysis was performed in 5.3% of patients (n 4) using a suspension, in 10.5% (n 8) using a smear, and in 34.2% (n 26) using CD138+ cells. Analyses of suspensions and smears were performed for 10.5% of patients (n 8), for 13.2% (n 10) of suspensions and CD138+ cells, and for 14.5% (n 11) of smears and CD138-positive cells. Analyses of all three materials were available for 6.6% of patients (n 5). Cut-offs were set at 5% for gains and translocations and at 10% for deletions in suspensions and smears. For analyses with CD138-positive cells, cut-off values of 10% for translocations and 20% for copy number alterations were used. Patients were divided into standard, high-risk, and ultra-high-risk groups. High-risk R-ISS aberrations were defined as 4 copies or more of t(4;14), t(14;16), t(14;20), del(17p), and amp (1q21). The presence of two risk markers was defined as double-hit myeloma. High-risk IMS 2024 (HR-IMS24) was defined as the presence of del17p, the TP53 mutation, biallelic del(1p32), and monoallelic del(1p32) with gain/amp1q, or the combination of any of the following translocations: t(4;14) or t(14;16) with gain/amp1q and/or del(1p32).

### 2.3. Statistical Analyses

Statistical analyses were performed using IBM SPSS Statistics 29 (IBM Corporation, Armonk, NY, USA). Categorical data are reported as numbers (n) and percentages (%), and continuous data are reported as median and interquartile range (IQR). Statistical tests were performed using cross-tabulations and the chi-squared test to assess associations among categorical variables. Continuous data were tested for normal distributions using Kolmogorov–Smirnov and Shapiro–Wilk tests. Normally distributed variables were analyzed for possible associated factors using ANOVA tests, while the Mann–Whittney U test was used for non-normally distributed variables. Statistical significance was assumed when the *p*-value was less than 0.05. The time to event is presented using a Kaplan–Meier curve with the log-rank test.

## 3. Results

### 3.1. Patient Characteristics

Our monocentric retrospective study included 74 patients with a median age at diagnosis of 62 years (53–71 years), of whom 62.2% (n 46) were male. The median age at the start of treatment with daratumumab was 66 years (56.75–75 years). All patients had cytogenetic results. A high cytogenetic risk after R ISS was documented in 39.2% of patients (n 29), while 13.5% of patients had ≥2 HR markers. In the double-hit myeloma group, 90% (n 9) of patients exhibited amp1q combinations and only one patient exhibited a del17p and t(4;14) combination. The HR-IMS24 criteria were met in 32.4% (n 24) of our patients.

At the start of the multiple myeloma treatment, an R-ISS of III was described in 33.8% of cases, and 85.1% (n 63) had an abnormal light chain ratio, fulfilling the SLIM-CRAB criteria. Calcium levels were elevated in 6.8% (n 5) of patients, no hyper viscosity was observed, and renal dysfunction was observed in 47.3% (n 35) of patients. Bone lesions were found in more than half of the patients (74.3%, n 55). Most patients had received two previous lines of therapy (37.8%, n 28) and autologous SCT (59.7%, n 44). Table 1 shows the patient characteristics.

**Figure 1:**
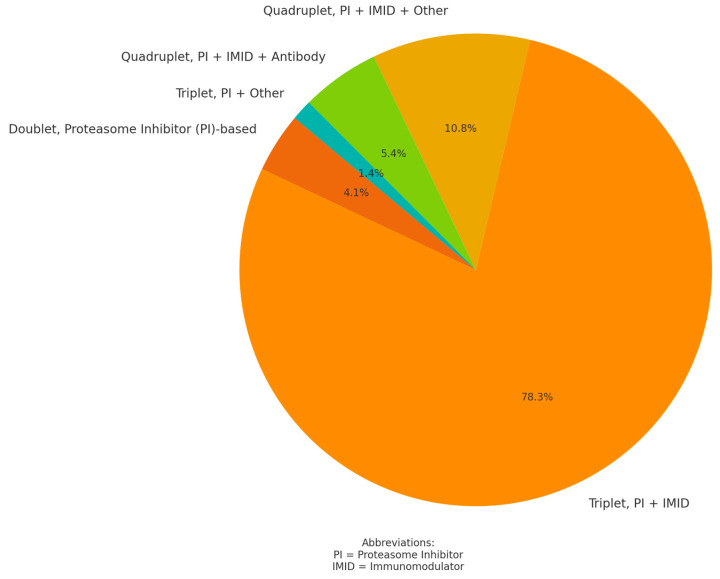
Previous myeloma treatment combinations (%).

Daratumumab was mostly used in the third line, with most patients (78.4%, n 58) having received a previous regimen of a proteasome inhibitor and an immunomodulator in triple combination with dexamethasone. Only three patients had received a double combination. A detailed overview of prior therapy combinations is shown in Figure 1. Treatment with the CD38 antibody daratumumab was initiated at a median of 36.52 months (14.31–72.93 months) after initial diagnosis.

**Table 1:** Patient characteristics.

		Number of Patients	Percentage
		74 (n)	100%
Gender	Male	46	62–2.1
	Female	28	37.8
Median age		62 yrs (53–71 IQR)	-
Median age at daratumumab initialization		66 yrs (56.75–75 IQR)	-
Median follow-up		5 yrs (3–7 IQR)	-
Patient deaths	Total number	29	39.4
	Male	18	62.1
	Female	11	37.9
Cause of death	Disease related	28	96.55
	Not disease related/unknown	1	8.45
Revised International Staging System	I	17	23.0
	II	32	43.2
	III	25	33.8
Myeloma subtype	IgG	34	45.9
	IgM	1	1.4
	IgA	20	27
	IgD	2	2.7
	Light-chain myeloma	17	22.97
	Kappa	47	63.51
	Lambda	27	36.49
Cytogenetic aberration	del17p	7	9.5
	t(4;14)	16	21.6
	amp1q21	26	35.1
	gain1q	17	23
	t(14;20)	0	0
	t(14;16)	0	0
	Del1p32	3	4.1
Cytogenetic risk	High-Risk R2-ISS	29	39.2
	Double-Hit R2-ISS	10	13.5
	High-Risk IMS2024	24	32.4
Previous lines of therapy	0	9	12.2
	1	17	23
	2	28	37.8
	3 or more	20	27
Previous stem cell transplant	Autologous median age	44	59.7
	Allogeneic	1	2.2
	No stem cell transplant	25	33.8
	Not available	1	2.2
Plasma cell infiltration	≥60%	20	27
	<60%	50	67.6
	Not available	4	5.4

Table 1: Patient characteristics at the time of initial diagnosis: Categorical variables are expressed as numbers and percentages. Continuous parameters are described in years (yrs) and interquartile range (IQR). High-risk R2ISS is defined as del(17p)/TP53 mutation, t(4;14), t(14;16), t(14;20), and amp1q. A ’double hit’ is defined as the presence of two of these high-risk R2ISS markers. High-risk IMS 2024 is defined as the presence of del17p, the TP53 mutation, biallelic del(1p32), monoallelic del(1p32) with gain/amp1q or the combination of any of the following translocations: t(4;14) or t(14;16) with gain/amp1q and/or del(1p32).

Figure 1: This graph shows the percentage distribution of previous therapies. Combinations, i.e., doublets/triplets and quadruplets, are listed. Proteasome inhibitors, immunomodulators, and antibodies are listed as combination partners. The most commonly used antibody was CD38; other combination therapies for myeloma, such as chemotherapy, are listed under “other”.

OS was significantly longer in patients who received a triplet combination in the first line than in those who received a doublet combination, at 63.54 (34.84–86.8) vs. 20.75 (7.81–53.74) months (*p* = 0.033).

### 3.2. Daratumumab Treatment

Daratumumab therapy was administered for an average of eight months (3–22 months). Treatment was discontinued in 87.7% (n 65) of patients and continued in nine patients. The reason for discontinuation was progression in 37 patients (50%) and death in 14 patients (18.9%). In the other 14 patients, de-escalation of myeloma treatment was the doctor’s decision due to toxicity or the patient’s response. Of the 14 patients with daratumumab discontinuation, 6 patients experienced progression during follow-up after a median time to relapse of 33.38 months (8.9–64.5 months). Discontinuation of therapy was not significantly associated with the occurrence of leukopenia or anemia at the last follow-up (62.24 months: 30.24–85.42 months). At the last follow-up, 29 patients (39%) had died.

**Figure 2:**
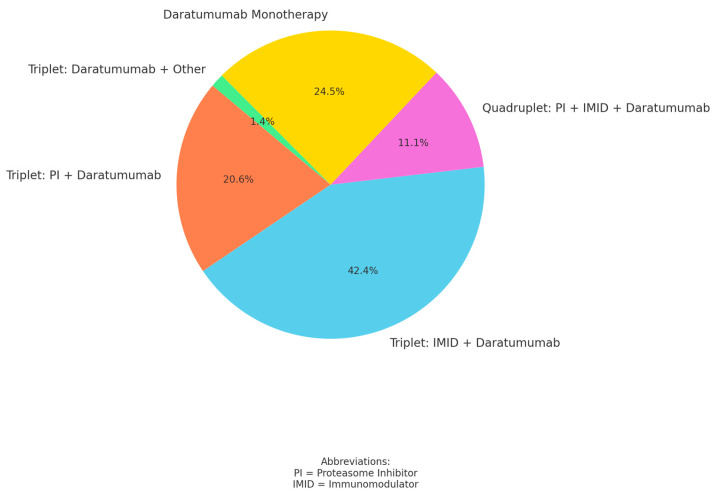
Different treatment combinations with daratumumab (%).

Most patients received combination therapy, most commonly with immunomodulators (41.9%, n 31). Only 24.3% (n 18) of patients received daratumumab monotherapy (Figure 2). The median DOT was 8 (3.07–22.01) months. Progression-free survival in the overall cohort was 15.52 (4.33–26.23) months, and the median overall survival was 64 (30.5–80) months.

Figure 2: This figure shows how daratumumab was administered. The different doublet, triplet, and quadruplet regimens with dexamethasone as the combination partner are shown as percentages.

Combination therapy resulted in a longer treatment duration than monotherapy (11.48 (3.49–21.49) months vs. 4.5 (2–23.06) months), which was not statistically significant (*p* = 0.415). In addition, PFS was prolonged in patients receiving combination therapy compared to those receiving monotherapy, again without statistical significance (17.02 months (7.01–26.94 months) vs. 4.5 months (2.33–24.05 months); *p* = 0.072).

During treatment with daratumumab, bone marrow infiltration decreased from a median of 42.5% (IQR 3.75–70%) at initial diagnosis to 1% (IQR 0–22.5%) after treatment with daratumumab.

### 3.3. Daratumumab in Cytogenetic Subgroups

In the study cohort, amp1q was detected in 35.1% (n = 26), gain1q in 23% (n = 17), t(4;14) in 21.6% (n = 16), del(1p32) in 4.1% (n = 3), and del17p in 9.5% (n = 7) of patients, t(14;16) and t(16;20) were not detected in any patient during the course of the disease prior to CD38 antibody treatment.

Neither amp1q (*p* = 0.347), t4;14 (*p* = 0.217), nor del 17p (*p* = 0.072) nor the presence of any of these poor prognostic aberrations in general (*p* = 0.174) had a significant impact on PFS. However, having double-hit myeloma significantly affected PFS, with a median of 17.34 months (8.62–17.58 months) in standard-risk (SR) patients compared to 6.24 months (1.36–16.35 months; *p* = 0.044) in double-hit patients. In addition, the PFS of patients who met the HR-IMS24 criteria was significantly shorter at 7.03 months (2.7–18.09 months, *p* = 0.023).

Similar results were obtained in the OS analyses. There was no significant impact on OS for patients with a high risk of R-ISS compared to SR patients (67.02 months: 49.5–101.7 months; 55.32 months: 27.61–85.57 months, *p* = 0.496), while there was a significant impact on OS for double-hit patients and patients meeting the HR-IMS24 criteria (42 months: 25.9–74.6 months, *p* = 0.035; 40 months: 21.14–74.47 months, *p* = 0.01). The seven patients without any abnormalities at initial diagnosis had a significantly longer OS compared to patients with cytogenetic abnormalities (82 months: 36–179.5 months, *p* = 0.007 vs. 59 months: 29.9–85.1 months), whereas of the 67 patients with genetic abnormalities at the time of initial diagnosis, 32 (47.8%) had a standard risk.

**Figure 3:**
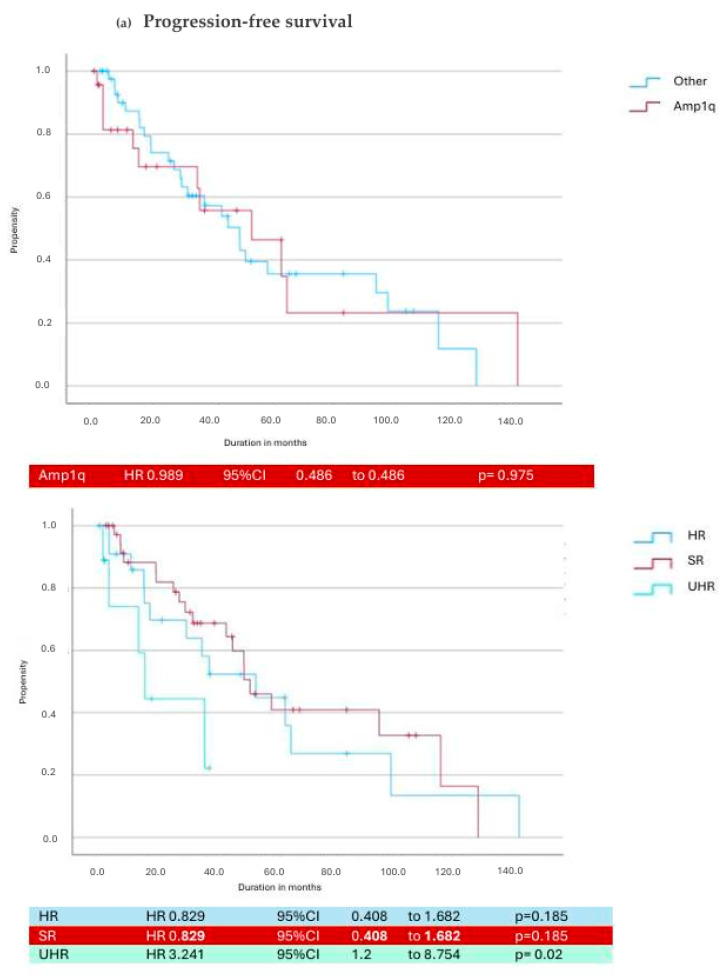

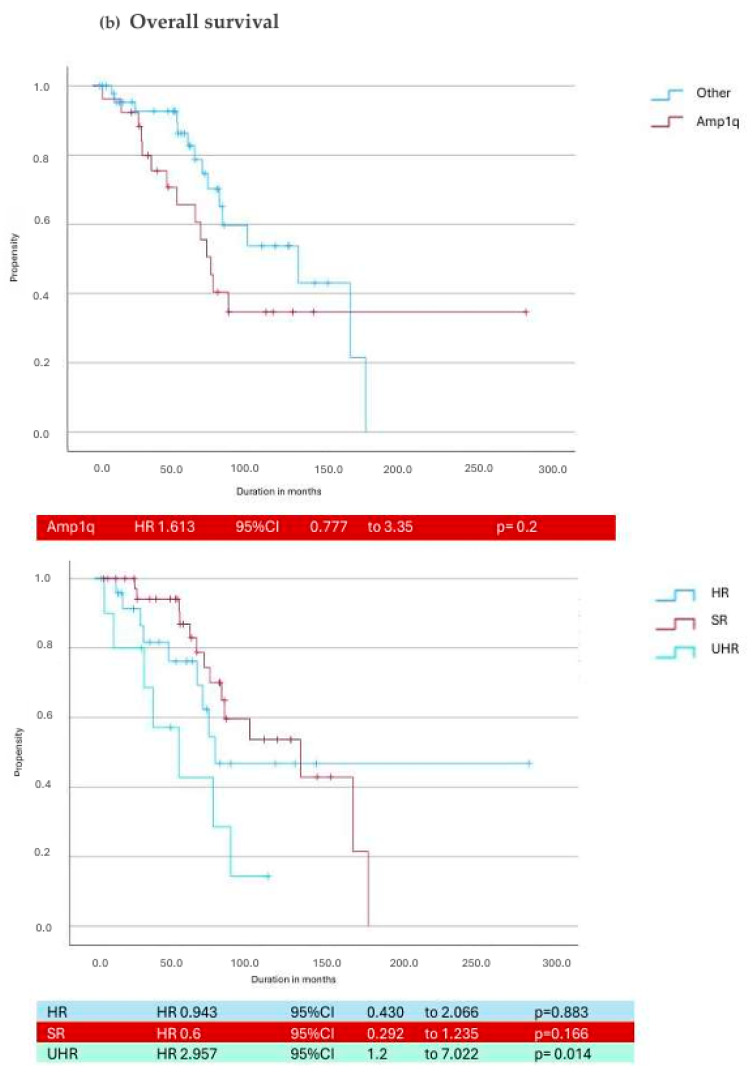
Survival curves and hazard ratios according to cytogenetic status.

Considering DOT, there was no difference between SR and HR patients (10.28 months: 3.5–22.01 months; 10.48 months: 3.1–25.8 months; *p* = 0.634) and individual HR markers (amp1q *p* = 0.811; t4;14: *p* = 0.175, del17: *p* = 0.624). The treatment duration was shorter (without statistical significance) in double-hit and HR-IMS24 patients (6.09 months: 1.1–16.5 months, *p* = 0.235; 5.16 months: 2–15.01 months, *p* = 0.108). Figure 3 shows the PFS and OS curves by cytogenetic abnormalities. PFS and OS data are shown in Table 2.

Figure 3: Survival curves and hazard ratios for progression-free (a) and overall (b) survival. Patients with amplification 1q (Amp1q) are shown as having a single cytogenetic alteration. In addition, the difference between standard-risk (SR) and high-risk cytogenetics (HR) and double hit (UHR) is shown. In the UHR group, 100% of patients had amp1q in addition to other high-risk markers. Hazard ratios, confidence intervals (95%CI), and *p*-values are shown.

**Table 2:** Cytogenetic risk and cytogenetic aberrations in relation to PFS, OS, and DOT. All *p*-values < 0.05 were considered statistically significant and are marked with *.

Cytogenetic Risk	Median PFS (Months)	Median OS (Months)	Median Duration of Treatment (Months)
**Standard Risk**	17.3 (8.6–27.6)	67 (49.5–101)	10.28 (3.5–22)
**High-Risk R2ISS**	13.1 (3.3–26.4)	55.3 (27.6–85.6)	10.48 (3.17–25.8)
**Double Hit**	6.2 (1.4–16.4)	42.8 (25.9–74.6) *	6.1 (1.1–16.5)
**High Risk IMS 2024**	7 (2.7–18.1) *	40.12 (21.1–74.5) *	5.2 (2–15)
**Amp 1q**	7.03 (1.95–22.4)	61.2 (32–83.8)	8 (2.5–24)
**Gain 1q**	14 (1.8–21.4)	53 (38.7–69.2)	11.5 (1.9–21.8)
**T(4;14)**	8.1 (4.5–19.1)	44 (22.4–63)	6.1 (1.7–19.1)
**Del17p**	5.7 (1.1–16)	17.18 (6.2–77.5)	5.03 (0.41–40.7)
**Del(1p32)**	13.4 (5.7–14.6)	40.7 (15–77.6)	10 (5.03–13)

Table 2: This table shows progression-free survival (PFS, defined as the time from start of daratumumab treatment to relapse/last follow-up or death), overall survival (OS, defined as the time from initial diagnosis to last follow-up or death) and duration of treatment (DOT) in median months (interquartile range). The results were stratified according to cytogenetic risk: high risk (t(4;14), t(14;16), t(14;20), del (17p) and amp1q(1q21)); double hit (when two risk markers occur simultaneously); or standard risk (including no cytogenetic abnormalities). High-risk IMS 2024 included patients with del17p, the TP53 mutation, biallelic del(1p32), or monoallelic del(1p32) with gain/amp1q or the combination of one of the following translocations: t(4;14) or t(14;16) with gain/amp1q and/or del(1p32).In addition, the results showed differentiations between individual risk aberrations: amplification 1q and the presence of another single high-risk aberration. All *p*-values < 0.05 were considered statistically significant and are marked with *.

The reduction in plasma cell infiltration (6%, 1.5–30%) in the bone marrow before and after daratumumab did not differ among cytogenetic risk groups, gender, or age. However, patient data were limited (n = 19).

### 3.4. Next Line of Treatment

After treatment with daratumumab, CD38 antibody treatment was restarted in 10 patients, starting at a median interval of 14.59 months (6.28–32.28 months). The median duration of re-treatment was 9 (2.5–17.7) months. The majority of patients received multiple lines of therapy (39.2% (n 29) ≥ five lines of therapy; 20.3% (n 15) two or three lines of therapy; 18.9% (n 14) four lines of therapy); only one patient received one line of therapy (1.4%). HR cytogenetics did not influence the number of lines of therapy (*p* = 0.907), even when considering amp1q (*p* = 0.597) and UHR patients (*p* = 0.338).

The time to re-treatment with daratumumab was not correlated with cytogenetic abnormalities, although the number of patients in this setting was limited (n = 10).

Table 3: Odds ratios, *p*-values, and 95% confidence intervals for factors influencing an event at 5 years are shown in Table 2. An event is defined as death or loss to follow-up and is considered overall survival in this analysis. Double-hit myeloma is defined as the presence of two high-risk aberrations (t(4;14), t(14;16), t(14;20), del (17p), and amp1q (1q21)).

## 4. Discussion

This retrospective, monocentric study provides important real-world data on the efficacy of daratumumab in patients with solitary amp1q and/or complex cytogenetic aberration patterns.

Daratumumab’s complex mechanism of action depends on several factors: direct cytotoxicity through the Fc region, antibody-dependent cellular cytotoxicity that is highly dependent on T cells (including NK cells), and complement-dependent cellular cytotoxicity that is inhibited by complement inhibitory proteins such as CD 55, 46, and 59 [17]. An impaired efficacy due to a reduction in natural killer cells, as well as due to an upregulation of complement inhibitory proteins, has been posited [18,19]. In patients with amp/gain1q, upregulation of CD55 is observed and has been discussed as a cause of the reduced response to daratumumab [13]. However, the complex immunomodulatory effect of daratumumab should be included in this discussion, as increased T cell activity, especially of CD8 cells, increased cytokine production with increased T cell activation, and increased T cell-mediated cytotoxicity have also been described [20].

The combination of daratumumab with an immunomodulatory drug such as lenalidomide may have a positive effect. Immunomodulators increase the activity and cytotoxicity of NK cells and may therefore act synergistically [21]. This is supported by our data showing that daratumumab combination therapy is associated with a significantly improved outcome.

Our data demonstrated a worse progression-free survival and overall survival in patients with the 1q aberration, but without statistical significance. This is in contrast to the work of Mohan et al., who showed significantly worse progression-free survival in RRMM patients, but this is in line with the previous findings of Parrondo et al. [15,22]. A systematic review by Neupane et al. discussed this issue and highlighted the heterogeneity of results. The main finding of this review is that effective treatments for multiple myeloma patients also work in the subgroup of patients with 1q aberrations. The review further highlights the efficacy of isatuximab and discusses the lack of data on daratumumab in this setting [6]. Previous results for isatuximab in ICARIA and IKEMIA found an improved PFS in high-risk patients with amp1q [16,23], and the first-line Perseus study presented encouraging data at EHA 2024 for DaraVRd in the subgroup of amp1q patients [1]. Our conclusion from our limited analysis and previous data is that daratumumab is an effective treatment that also works in amp/gain1q patients.

However, our data highlight the negative impact of the co-occurrence of amp1q with other high-risk markers in a double-hit pattern, which was also described by Lim et al. [24]. This pattern leads to poorer outcomes with high-intensity first-line treatment. In our study, this subgroup exhibited a statistically significant reduction in PFS and OS. The negative effect of amp1q in combination with other high-risk markers can therefore be confirmed. This effect was not overcome by the addition of daratumumab alone in our cohort.

Proposed treatments for these double-hit patients have been evaluated in the Concept trial, for example. However, the new IMS 2024 criteria have not yet been included in these data. Thus, it remains to be discussed whether these results are transferable to the new risk criteria. In any case, our analysis has shown that these patients have similarly impaired outcomes, which is an interesting finding for clinical practice. Both progression-free survival and overall survival are limited, whereas such limitations were not observed in patients meeting the old R-ISS criteria. This highlights the fact that, with improved treatment options, our high-risk patients can now be better identified using the more stringent risk criteria agreed upon at IMS 2024 [14].

Double-hit patients, as shown in the recent MASTER trial with daratumumab and the CONCEPT trial with isatuximab, carfilzomib, lenalidomide, and dexamethasone, remain a special patient population that needs to be considered in a different manner and may relapse earlier, even with intensive treatment. Thus, intensive continuous treatment concepts or the early use of new treatment options such as T-cell-engaging therapies should be considered [9,25].

Our patient characteristics are comparable in terms of age to previous studies, such as Castor, with a similar follow-up of over five years. Patients were predominantly of transplantable age at initial diagnosis with primary triplet induction. In terms of frontline treatment, triplet combinations and ASCT had the expected effect, as recently underlined by the long-term data from the Determination study [26].

The limitations of our study should be mentioned. The analysis was retrospective. However, this allows for an analysis of the actual data without the bias of exclusion criteria and intensive controls. In addition, the limited number of patients and the use in a relapsed setting should be mentioned. However, this allowed for a long follow-up period and, therefore, a calculation of the impact on PFS/OS. Response rates were not reported, but PFS, OS, and duration of treatment are the main treatment outcomes in relapsed multiple myeloma and, therefore, the most important outcome parameters. In addition, this retrospective analysis showed a relative accumulation of high-risk markers. This is explained by the negative selection, since high-risk patients relapse earlier and are more likely to require relapse treatments. In addition, the source of the risk is more likely to be treated at a hematology center.

## 5. Conclusions

Our analyses provide extensive real-world data on the robust effect of daratumumab on cytogenetic aberrations, particularly amp1q. However, double-hit patients and patients meeting the high-risk criteria of IMS 2024 remain a difficult-to-treat cohort that cannot be adequately managed by the addition of the anti-CD38 antibody daratumumab alone. Therefore, new therapeutic approaches are urgently needed in this patient population.

## Figures and Tables

**Table 3:** Multivariate analyses of factors influencing 5-year overall survival in patients with relapsed/refractory myeloma treated with daratumumab.

	sOR	95% Confidence Interval		*p*-Value
		Lower Value	Upper Value	
**Sex**	1.1	0.39	2.88	0.905
**Age**	0.38	0.92	1.56	0.379
**SCT**	1.25	0.35	4.47	0.727
**Double hit myeloma**	5.24	1.01	27.24	0.049

Abbreviations: SCT—stem cell transplantation.

## Data Availability

The data that support the findings of this study are available from the corresponding author, [mb], upon reasonable request.
